# Differences in Cardiac Output and Aerobic Capacity Between Sexes Are Explained by Blood Volume and Oxygen Carrying Capacity

**DOI:** 10.3389/fphys.2022.747903

**Published:** 2022-03-17

**Authors:** Candela Diaz-Canestro, Brandon Pentz, Arshia Sehgal, David Montero

**Affiliations:** ^1^Libin Cardiovascular Institute of Alberta, University of Calgary, Calgary, AB, Canada; ^2^Faculty of Kinesiology, University of Calgary, Calgary, AB, Canada; ^3^Cumming School of Medicine, Calgary, AB, Canada

**Keywords:** blood volume, hemoglobin mass, cardiac function, aerobic capacity, sex

## Abstract

Whether average sex differences in cardiorespiratory fitness can be mainly explained by blood inequalities in the healthy circulatory system remains unresolved. This study evaluated the contribution of blood volume (BV) and oxygen (O_2_) carrying capacity to the sex gap in cardiac and aerobic capacities in healthy young individuals. Healthy young women and men (*n* = 28, age range = 20–43 years) were matched by age and physical activity. Echocardiography, blood pressures, and O_2_ uptake were measured during incremental exercise. Left ventricular end-diastolic volume (LVEDV), stroke volume (SV), cardiac output (Q), peak O_2_ uptake (VO_2p*eak*_), and BV were assessed with precise methods. The test was repeated in men after blood withdrawal and reduction of O_2_ carrying capacity, reaching women’s levels. Before blood normalization, exercise cardiac volumes and output (LVEDV, SV, Q) adjusted by body size and VO_2p*eak*_ (42 ± 9 vs. 50 ± 11 ml⋅min^–1^⋅kg^–1^, *P* < 0.05) were lower in women relative to men. Blood normalization abolished sex differences in cardiac volumes and output during exercise (*P* ≥ 0.100). Likewise, VO_2p*eak*_ was similar between women and men after blood normalization (42 ± 9 vs. 40 ± 8 ml⋅min^–1^⋅kg^–1^, *P* = 0.416). In conclusion, sex differences in cardiac output and aerobic capacity are not present in experimental conditions matching BV and O_2_ carrying capacity between healthy young women and men.

## Introduction

Peak oxygen uptake (VO_2p*eak*_) per kg of body weight, a hallmark of aerobic exercise capacity, is on average ∼15–25% lower in women relative to men with similar training status, although substantial overlap exists (Rusko, 1992; Lundby and Robach, 2015). In theory, multiple variables along the O_2_ transport and utilization chain might potentially explain such a sex dimorphism in VO_2p*eak*_ (Lundby et al., 2017). Indeed, inherent sex differences can be found in the structure and function of the lungs, blood, heart, arteries, skeletal muscle fibers and mitochondria, where O_2_ is finally consumed (Pfaffenberger et al., 2013; Lundby and Robach, 2015; Joyner et al., 2016; Sheel et al., 2016; Regitz-Zagrosek and Kararigas, 2017; Montero et al., 2018). The step down the O_2_ cascade that ultimately limits VO_2p*eak*_ in women and thus primarily explain this sex dimorphism has long remained speculative (Charkoudian and Joyner, 2004; Diaz-Canestro and Montero, 2019, 2020).

On the basis of cumulative evidence since the inception of modern exercise physiology (Kjellberg et al., 1949; Dill et al., 1974; Brotherhood et al., 1975; Heinicke et al., 2001; Levine, 2008; Lundby and Robach, 2015; Montero et al., 2015, 2016a,2016b,^2017^; Lundby et al., 2017; Montero and Lundby, 2017a,b, 2018), we recently tested the contribution of two definite blood variables in an older cohort (mean age = 64 ± 8 years, range = 42–88 years) (Diaz-Canestro et al., 2021). Blood volume (BV) and O_2_ carrying capacity were experimentally manipulated in men to match women’s levels (Diaz-Canestro et al., 2021). As a result, the large sex difference in VO_2p*eak*_ was precisely abolished (Diaz-Canestro et al., 2021). Yet, key central determinants of O_2_ delivery, i.e., cardiac filling, stroke volume (SV) and output (Q), remained elevated during exercise in men (relative to women) after blood normalization (Diaz-Canestro et al., 2021). In parallel, cardiac afterload was reduced in men following blood normalization, plausibly facilitating venous return, cardiac filling, and Q at high exercise intensities due to additional peripheral vasodilation induced by lowered blood O_2_ content, which in turn may affect blood flow distribution and O_2_ extraction (Calbet et al., 2006; Lundby et al., 2008; Casey and Joyner, 2012; Diaz-Canestro et al., 2021). Taken together, the sex gap in VO_2p*eak*_ in the older cohort seems to be explained by the interaction of BV and O_2_ carrying capacity with hemodynamic and molecular regulatory mechanisms that determine optimal delivery of O_2_ via the circulatory system. Whether these findings can be extrapolated to healthy young individuals with a circulatory system not affected by the inexorable effects of aging remains unknown. Specifically, could BV and O_2_ carrying capacity explain sex differences in cardiorespiratory fitness at the period of life in that feats of endurance performance are achieved? The answer to such question will provide relevant and plausibly sensitive insight since the aforementioned blood characteristics are amenable to lifestyle as well as pharmacological modification (Jelkmann and Lundby, 2011; Lundby and Robach, 2015; Montero et al., 2015, 2017; Montero and Lundby, 2017a).

This study aimed to experimentally assess the impact of sex differences in BV and O_2_ carrying capacity on cardiac function, central hemodynamics, and O_2_ uptake during incremental exercise in healthy young individuals. It was hypothesized that the match of BV and O_2_ carrying capacity in young men and women would eliminate the sex gap in cardiac as well as aerobic capacities.

## Materials and Methods

### Participants

Twenty-eight healthy young adult women and men (< 44 years) were recruited via electronic/printed advertisements on community notice boards in the city of Calgary. Moderate-to-vigorous physical activity (MVPA) levels were determined from established questionnaires as previously described (Montero et al., 2016c). Inclusion criteria comprised healthy status, absence of current medical symptoms or medication limiting incremental exercise testing, and no history of cardiac, pulmonary, or neuromuscular diseases. Individuals fulfilling the above criteria but having donated blood within 3 months prior to the study were excluded. The study was approved by the Conjoint Health Research Ethics Board (REB18-1654) of the University of Calgary and conducted in accordance with the declaration of Helsinki. Prior to the start of the experiments, informed oral and written consents were obtained from all participants.

### Experimental Design

Participants were required to report to our laboratory at least once, depending on sex and a voluntary familiarization visit. Each man was assessed twice, prior to and after blood normalization relative to a previously assessed woman with similar age and physical activity level (one-to-one matching). Time of day of testing sessions was kept consistent for each men and women–men matched pair with a minimum of 48 h and a maximum of 7 days between the first (baseline) and second (blood normalization) sessions. All individuals were instructed to avoid strenuous exercise, alcohol and caffeine from 24 h prior to testing, as well as to maintain their usual baseline activity and daily dietary habits throughout the study. All measurements were performed after a 5-h fasting period in a quiet room with controlled temperature between 22 and 23°C. Prior to testing the participants completed demographic and clinical questionnaires and rested in supine position for 20 min in order to stabilize cardiovascular, hemodynamic and hematological variables.

### Experimental Measures

#### Hemoglobin Mass (Hb_*mass*_) and Blood Volumes

Hemoglobin mass (Hb_*mass*_) was determined using the classic carbon monoxide (CO) rebreathing technique integrated in a semi-automated system with a very low typical error of measurement (TE ≤ 1.2%), as previously described (Montero et al., 2015, 2017; Siebenmann et al., 2017). In brief, following 20 min of supine rest, 2 ml of blood (baseline) was sampled from the median cubital vein via a 20-G venflon (BD, United States) and analyzed immediately in duplicate for percent carboxyhemoglobin (%HbCO), hemoglobin (Hb) concentration and hematocrit (Hct) (ABL80, Radiometer, Denmark). Individuals performed the aerobic capacity test with unaltered hematological variables in the first testing session. After the aerobic capacity test was completed and following 20 min of supine recovery, they breathed 100% O_2_ for 4 min to flush the nitrogen from the airways. After closing the O_2_ input, a bolus of 1.5 ml/kg of 99.5% chemically pure CO (Air Liquide, Canada) was administrated into the breathing circuit. Individuals rebreathed this gas mixture for 10 min. Then, an additional 2 ml blood sample was obtained and analyzed in duplicate as aforementioned. The change in%HbCO is used to calculate Hb_*mass*_, taking into account the small amount of CO that remains in the rebreathing circuit at the end of the procedure. Total red blood cell volume (RBCV), plasma volume (PV), and blood volume (BV) were determined from Hb_*mass*_, baseline Hb concentration and baseline Hct (Montero et al., 2015, 2017; Siebenmann et al., 2017).

### Transthoracic Echocardiography and Central Hemodynamics

Apical four-chamber and two-chamber cine-loops were continuously recorded via high-resolution ultrasound (Mindray Medical M9, United States) and analyzed offline (TOMTEC Imaging Systems, Royal Philips, Netherlands) at rest and during predetermined levels of incremental exercise relative to peak heart rate (HR_*peak*_) (60, 70, 80, 90, and 100% HR_*peak*_) as well at a given submaximal workload (100 W). Following the American Society of Echocardiography and the European Association of Cardiovascular Imaging recommendations, two-dimensional (2D) cardiac chamber quantification was performed using the modified Simpson method (biplane method of disks) by tracing the endocardial border in both apical four-chamber and two-chamber views at end-diastole and end-systole (Pellikka et al., 2007; Lang et al., 2015). Systolic blood pressure (SBP), diastolic blood pressure (DBP), and mean arterial pressure (MAP) at the heart level were continuously assessed non-invasively via Finometer PRO (Finapres Medical Systems, Netherlands) (Schutte et al., 2004), with data exported into a pre-established acquisition software (Labchart 7, AD Instruments, United Kingdom). Stroke volume (SV) was determined as left ventricular end-diastolic volume (LVEDV) minus left ventricular end-systolic volume (LVESV), while the product of SV and HR provided cardiac output (Q). It should be noted that 2D echocardiography intrinsically underestimates SV and Q due to the geometry of the heart (Patel et al., 2021), yet its high temporal resolution and reliability is required to obtain precise images in a given cardiac cycle during exercise. The Fick equation was used to assess the effect of blood normalization on peak arteriovenous O_2_ difference, a variable that was not directly measured but estimated by the ratio of VO_2p*eak*_ and Q_*peak*_. Systemic vascular resistance (SVR) was calculated as the ratio of MAP and Q. Total arterial compliance was determined by the pulse pressure (PP) method (SV/PP) (Chemla et al., 1998). Echochardiographic variables are commonly normalized by body surface area (BSA = 0.007184⋅weight^0.425^⋅height^0.725^) (Du Bois and Du Bois, 1989). The reproducibility of key echocardiographic and hemodynamic measurements [within-subject coefficient of variation (CV)] during incremental exercise in our laboratory is ≤ 6% for left ventricular (LV) volumes, ≤ 3% for blood pressures and ≤ 7% for SVR.

#### Aerobic Capacity

An established incremental exercise protocol (Montero et al., 2015, 2017; Montero and Lundby, 2017b) was performed using an electromagnetic cycle ergometer (KICKR Core trainer, Wahoo, United States) integrated within a large lower body negative pressure chamber (LBNP) (165 × 82 × 108 cm) designed for exercise echocardiography (Tymko SB, Canada). The LBNP comprises an electric hydraulic jack that enables to select any degree from 0 to 45^°^ of left lateral tilting (Supplementary Figure 1). The combination of left semilateral supine body position (17° relative to the horizontal) with lower body negative pressure allows for the simultaneous assessment of cardiac function—which requires a left lateral body position for high-quality and reproducible imaging—and aerobic capacity via the regulation of negative pressure inside the chamber (–50 mmHg) to induce hemodynamic loads characteristic of the upright position, a physiological requirement to achieve VO_2p*eak*_ (Murthy et al., 1994; Boda et al., 2000). The test started with a warm-up period of 3 min at 20–30 W workloads. Thereafter, the workload was increased by 15–30 W every 60 s until exhaustion was reached in a total duration of 7–10 min. O_2_ uptake and CO_2_ output were continuously measured (CardioCoach VO_2_, KORR Medical, United States). Calibration of the gas analyzers and the flowmeter was performed prior to each test. Breath-by-breath values were averaged over 15s (Martin-Rincon and Calbet, 2020). The highest breath-by-breath average value was taken as VO_2p*eak*_ provided that two of the following established criteria were fulfilled: Plateau in O_2_ uptake despite increased workload, age-predicted HR_*peak*_ ± 10 bpm (HR_*peak*_ = 211–0.64 × age) (Nes et al., 2013), respiratory exchange ratio ≥ 1.1 (Montero et al., 2015; Kaminsky et al., 2017).

#### Blood Normalization

BV and O_2_ carrying capacity were reduced in men to the same levels of women with similar age and physical activity level (one-to-one matching) in the second testing session. The opposite approach, i.e., to increase these variables in women to the level of men was not implemented due the convoluted and uncertain methodology related to autologous blood transfusion. In order to match BV in the second testing session, a 20 G venflon (BD, United States) was placed in the median cubital vein and a certain amount of blood (8.1 ± 2.3 ml⋅kg^–1^, ranging from 0 to 11.3 ml⋅kg^–1^) was withdrawn immediately before starting the measurements, which resulted in identical BV per kg between men and women. Hemoconcentration was not influenced by this procedure, Hb concentration and Hct were unaltered (*P* ≥ 0.816) O_2_ carrying capacity was defined as the concentration in blood of Hb able to carry O_2_ [i.e., effective Hb (g⋅dl^–1^) = total Hb (g⋅dl^–1^)–(HbCO (g⋅dl^–1^) + methemoglobin (g⋅dl^–1^)]. Accordingly, a small quantity of CO, determined by the difference in effective Hb between men and women, was introduced in the rebreathing system in which men breathed for 10 min in order to reduce their O_2_ carrying capacity to women’s level. The level of effective Hb was monitored prior to as well as right after exercise testing in each men via venous blood sampling to control and corroborate the reduction of blood O_2_ carrying capacity to the desired levels (Christensen et al., 1993; Gonzalez-Alonso et al., 2001; Montero and Lundby, 2019). During the blood normalization procedure, men rested in supine position with their lower body inside the LBNP-exercise chamber.

### Statistical Analysis

Statistical analyses were performed using SPSS 22.0 (SPSS, Chicago, IL). Data were tested for normal distribution with the Kolmogorov-Smirnov test and for homogeneity of variances with Levene’s test. Two-way ANOVA with repeated measures was performed to assess echocardiographic, hemodynamic, and pulmonary variables in women and men prior to and after blood normalization, with group (women, men prior to/after blood normalization) and time (60, 70, 80, 90, and 100% HR_*peak*_) as between- and within-subject factors, respectively. When F was significant in the ANOVA, pair-wise specific comparisons were carried out via independent sample *t*-tests. Pair-wise specific comparisons were secondary and explorative in nature, thus they were not corrected for alpha inflation. A two-tailed *P*-value less than 0.05 was considered significant. All data are reported as mean (± SD) unless otherwise stated.

## Results

### General Characteristics

Table 1 present main baseline characteristics of the study subjects. Women had smaller anthropometric indices (height, weight, BSA) than men (*P* ≤ 0.006). Physical activity levels, determined by MVPA, did not differ between women and men (*P* ≥ 0.140). Key LV volumetric variables (LVEDV, LVESV, SV indexed by BSA) were lower in women (*P* ≤ 0.039), whereas HR was similar between sexes. Blood pressures at rest did not differ between sexes, whereas women presented with higher SVR and decreased total arterial compliance (*P* ≤ 0.028). Cardiorespiratory fitness, as represented by VO_2p*eak*_, was lower in women relative to men (*P* = 0.049).

**TABLE 1:** Baseline characteristics of study subjects.

	Women	Men
*N*	14	14
Age (yrs)	33 ± 6	29 ± 7
Height (cm)	169.0 ± 3.8	179.4 ± 9.9^*^
Weight (kg)	65.0 ± 5.9	78.7 ± 15.9^*^
BSA (m^2^)	1.7 ± 0.1	2.0 ± 0.2^*^
MVPA (h⋅wk^–1^)	6.0 ± 3.3	8.5 ± 5.2
Smoking (%)	0	0
**Resting echocardiography**		
HR (bpm)	62 ± 9	59 ± 9
RA (ml⋅m^–2^)	21.0 ± 9.5	20.1 ± 5.5
RV EDA (cm^2^⋅m^–2^)	12.0 ± 2.4	11.5 ± 1.9
RV ESA (cm^2^⋅m^–2^)	5.7 ± 1.2	4.9 ± 1.0
LA (ml⋅m^–2^)	20.7 ± 8.4	21.6 ± 6.3
LVEDV (ml⋅m^–2^)	45.5 ± 10.5	59.3 ± 14.2^*^
LVESV (ml⋅m^–2^)	12.4 ± 3.5	18.5 ± 6.9^*^
LVEF (%)	72.2 ± 7.9	69.1 ± 5.2
SV (ml⋅m^–2^)	33.1 ± 9.7	40.6 ± 8.6^*^
**Resting hemodynamics**		
SBP (mmHg)	131 ± 16	133 ± 19
DBP (mmHg)	74 ± 21	74 ± 13
MAP (mmHg)	88 ± 12	89 ± 18
SVR (dyn⋅s⋅cm^–5^)	2,108 ± 536	1,588 ± 558^*^
SV/PP (ml⋅mmHg^–1^)	1.1 ± 0.4	1.4 ± 0.4^*^
**Aerobic capacity**		
VO_2p*eak*_ (ml⋅min^–1^)	2,725 ± 549	3,862 ± 816^*^
VO_2p*eak*_ (ml⋅min^–1^⋅kg^–1^)	42 ± 9	50 ± 11^*^

*Data are presented as mean ± SD.*

*P < 0.05, women vs. men.

*BSA, body surface area; DBP, diastolic blood pressure; HR, heart rate; LA, left atria; LVEDV, left ventricular end-diastolic volume; LVEF, left ventricular ejection fraction; LVESV, left ventricular end-systolic volume; MAP, mean arterial pressure; MVPA, moderate-to-vigorous physical activity; PP, pulse pressure; RA, right atria; RV EDA, right ventricle end-diastolic area; RV ESA, right ventricle end-systolic area; SBP, systolic blood pressure; SV, stroke volume; SVR, systemic vascular resistance; VO_2peak_, peak oxygen uptake.*

### Blood O_2_ Carrying Capacity and Blood Volume

Hematological parameters in women and men before and after blood normalization are shown in Table 2. Blood O_2_ carrying capacity, characterized by effective Hb concentration, and Hct were lower in women compared with men (*P* < 0.001). Blood normalization matched effective Hb (11.9 ± 0.9 vs. 12.0 ± 0.8 g⋅dl^–1^, *P* = 0.696) and BV (86.2 ± 11.5 vs. 86.2 ± 10.2 ml⋅kg^–1^, *P* = 0.987) in women and men. The BV withdrawn from men was 8.1 ± 2.8 ml⋅kg^–1^, slightly superior to a standard blood donation (Rios et al., 2010).

**TABLE 2:** Hematological variables in women and men prior to and after blood normalization.

	Women	Men pre	Men post
Hb_*mass*_ (g)	655.8 ± 86.6	1,011.9 ± 133.8^*^	928.5 ± 144.9^†^
HbCO (%)	0.5 ± 0.2	0.7 ± 0.2	14.4 ± 4.0^†^
Effective Hb (g⋅dl^–1^)	11.9 ± 0.9	13.8 ± 0.6^*^	12.0 ± 0.8
Hct (%)	40.4 ± 3.0	47.2 ± 1.9^*^	47.3 ± 1.8^†^
RBCV (ml⋅kg^–1^)	34.8 ± 5.2	43.9 ± 5.1^*^	40.8 ± 4.9^†^
PV (ml⋅kg^–1^)	51.4 ± 7.6	49.3 ± 6.9	45.5 ± 5.7
BV (ml⋅kg^–1^)	86.2 ± 11.5	93.2 ± 11.5	86.2 ± 10.2

*Data are presented as mean ± SD.*

^*^P < 0.05, women vs. men pre.

*^†^P < 0.05, women vs. men post. BV, blood volume; Effective Hb, blood concentration of hemoglobin able to carry oxygen; HbCO, carboxyhemoglobin; Hb_mass_, total circulating hemoglobin mass; Hct, hematocrit; PV, plasma volume; RBCV, red blood cell volume.*

### Exercise Echocardiography and Hemodynamics During Exercise

**FIGURE 1:**
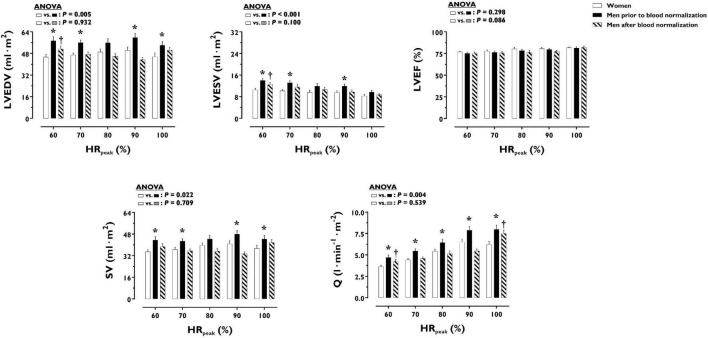
Left ventricular volumes and function during incremental exercise in women and men prior to and after blood normalization. *P < 0.05 between women and men prior to blood normalization. ^†^*P* < 0.05 between women and men after blood normalization. Data are expressed as mean ± SEM. Number of biological observations for each graph: LVEDV (*n* = 195), LVESV (*n* = 195), LVEF (*n* = 195), SV (*n* = 195), Q (*n* = 195). Echocardiographic data were analyzed within ± 5 bmp of specific percentages of HR_*peak*_. HR_*peak*_, peak heart rate; LVEDV, left ventricular end-diastolic volume; LVEF, left ventricular ejection fraction; LVESV, left ventricular end-systolic volume; Q, cardiac output; SV, stroke volume.

**FIGURE 2:**
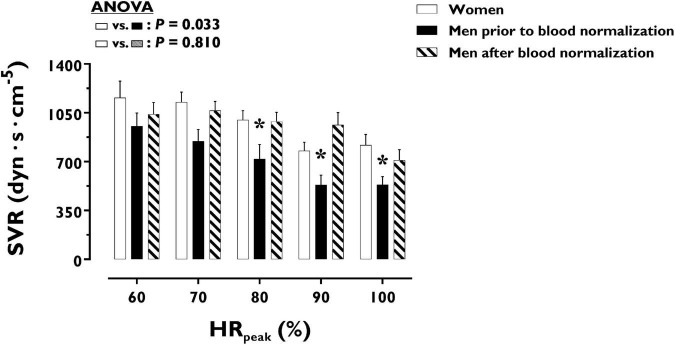
Systemic vascular resistance during incremental exercise in women and men prior to and after blood normalization. *P < 0.05 between women and men prior to blood normalization. ^†^*P* < 0.05 between women and men after blood normalization. Data are expressed as mean ± SEM. Number of biological observations in the graph: *n* = 145. Echocardiographic data were analyzed within ± 5 bmp of specific percentages of HR_*peak*_. HR_*peak*_, peak heart rate; SVR, systemic vascular resistance.

**FIGURE 3:**
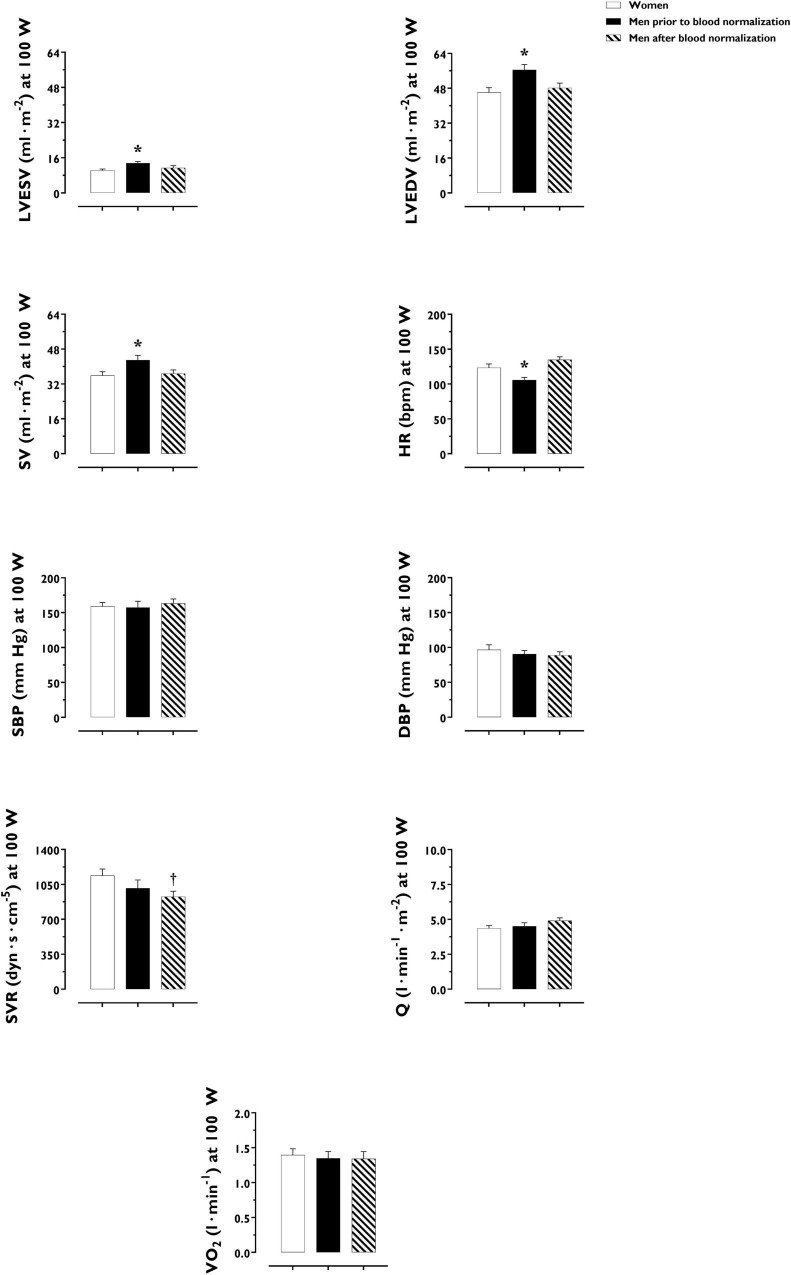
Cardiac, hemodynamic and pulmonary variables at a given fixed submaximal exercise workload (100 W) in women and men prior to and after blood normalization. *P < 0.05 between women and men prior to blood normalization. ^†^*P* < 0.05 between women and men after blood normalization. Data are expressed as mean ± SEM. Number of biological observations for each graph: LVEDV (*n* = 42), LVESV (*n* = 42), SV (*n* = 42), HR (*n* = 42), SBP (*n* = 38), SVR (*n* = 38), Q (*n* = 42), VO_2_ (*n* = 35). HR, heart rate; LVEDV, left ventricular end-diastolic volume; LVEF, left ventricular ejection fraction; Q, cardiac output; SBP, systolic blood pressure; SV, stroke volume; SVR, systemic vascular resistance; VO_2_, oxygen uptake (per kg of body weight).

LV volumes and function during incremental exercise are displayed in Figure 1. Prior to blood normalization, LV volumes (LVEDV, LVESV, SV) were lower in women compared with men (*P* ≤ 0.022). Consequently, women showed reduced Q at any given relative exercise intensity (*P* = 0.004). Blood normalization abolished sex differences in LV volumes and output (*P* ≥ 0.100). Women and men presented similar LVEF, an overall index of systolic function, irrespective of blood normalization (*P* ≥ 0.086). Likewise, sex differences were not observed in central blood pressures during incremental exercise prior to or after blood normalization (*P* ≥ 0.185). In contrast, SVR was higher in women relative to men before blood normalization (*P* = 0.007), a sex difference that was vanished after blood normalization (*P* = 0.810) (Figure 2). HR_*peak*_ was lower in women relative to men before blood normalization (170.1 ± 12.9 vs. 179.9 ± 9.4 bpm, *P* = 0.024) and was not altered after blood normalization in men (179.5 ± 9.2 bpm). Blood normalization induced a –13.0% reduction in estimated peak arteriovenous O_2_ difference in men. At a given absolute submaximal exercise workload (100 W) (Figure 3), women presented reduced LVEDV and SV (*P* ≤ 0.019) but higher HR (*P* = 0.013) compared with men prior to blood normalization, which resulted in similar Q (*P* = 0.656) between sexes. Blood normalization abolished sex differences in submaximal LV volumes and HR (*P* ≥ 0.109).

### Aerobic Capacity

**FIGURE 4:**
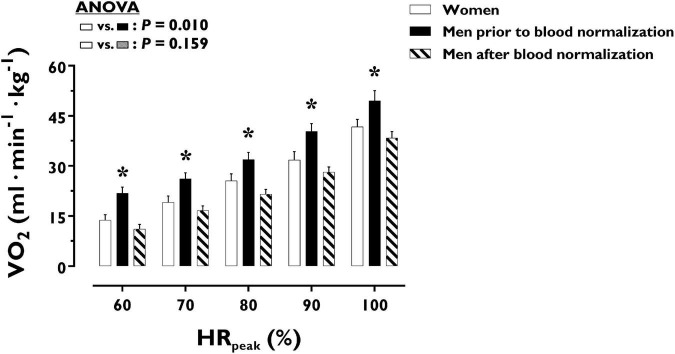
Oxygen uptake during incremental exercise in women and men prior to and after blood normalization. *P < 0.05 between women and men prior to blood normalization. ^†^*P* < 0.05 between women and men after blood normalization. Data are expressed as mean ± SEM. Number of biological observations in the graph: *n* = 210. Echocardiographic data were analyzed within ± 5 bmp of specific percentages of HR_*peak*_. HR_*peak*_, peak heart rate; VO_2_, oxygen uptake (per kg of body weight).

O_2_ uptake throughout incremental exercise is displayed in Figure 4. At any specific relative exercise intensity, O_2_ uptake was reduced in women compared with men before blood normalization (*P* = 0.010). After blood normalization, there was no sex difference in O_2_ uptake during exercise (*P* = 0.159). VO_2p*eak*_ was similar in women and men after blood normalization (42 ± 9 vs. 40 ± 8 ml⋅min^–1^⋅kg^–1^, *P* = 0.416). At an absolute submaximal exercise workload (100 W) (Figure 3), O_2_ uptake (l⋅min^–1^) did not differ in women and men before (*P* = 0.760) and after (*P* = 0.721) blood normalization.

## Discussion

The main purpose of this study was to experimentally determine the contribution of key hematological variables on sex differences in cardiorespiratory fitness in healthy young individuals. According to the initial hypothesis, blood normalization between women and men virtually eliminated sex differences in cardiac function, including key LV volumetric determinants of cardiac pumping capacity. Similarly, large sex differences in aerobic capacity were abolished after blood normalization. The present findings support the fundamental contribution of blood variables determining O_2_ delivery to cardiorespiratory fitness in healthy young individuals.

The connection between the most precious fluid in the human body and aerobic exercise capacity was first acknowledged in the aftermath of World War II (Kjellberg et al., 1949). Likewise, the weakening effects of blood donation on endurance athletes were already noticed in the 1940s when the Springfield College ruled that “*no man may place his name on the blood donors list while actively engaged in a varsity sport*” (Karpovich and Millman, 1941). Eight decades later, thanks to the progressive development of accurate methods to assess hematological, cardiovascular and metabolic systems at work, the underlying physiology is fundamentally understood. In the presence of normal lung function, cardiorespiratory fitness, as commonly represented by VO_2p*eak*_, is essentially, albeit not exclusively, determined by the circulatory capacity to deliver O_2_ (Levine, 2008; Lundby et al., 2017). Such a cardinal variable is not only a function of blood O_2_ carrying capacity (Lundby et al., 2017; Montero et al., 2019). Indeed, blood plays a fundamental role as a primary hemodynamic “driver” of the human circulatory system. The more blood fills the system, particularly the heart, the greater the output (Q = SV × HR), until a plateau is reached, conforming to the Frank-Starling mechanism or “Law of the heart,” as plainly formulated at the dawn of twentieth century (Maestrini, 1915; Pezza, 1974). The primary role of BV in determining peak cardiac and aerobic capacities, i.e., Q_*peak*_ and VO_2p*eak*_, has been unequivocally demonstrated by phlebotomy studies showing proportional reduction in BV, Q_*peak*_ and VO_2p*eak*_, which do not recover until BV is reestablished (Panebianco et al., 1995; Meurrens et al., 2016; Van Remoortel et al., 2017). Likewise, typical increments in Q_*max*_ and VO_2m*ax*_ (7–10%) induced by endurance training are reverted to pre training levels after negating training-induced gains in BV (Bonne et al., 2014; Montero et al., 2015), despite the presence of marked skeletal muscle adaptations in healthy young individuals (Montero et al., 2015). Yet, we cannot obviate the fact that the vast majority of available evidence supporting our current understanding arises from studies comprised of men (Levine, 2008; Lundby et al., 2017; Montero and Lundby, 2018). Would the average sex differences in cardiorespiratory fitness be explained by BV and effective Hb concentration? The current study experimentally demonstrates the complete elimination of a 19% sex gap in VO_2p*eak*_, which is by no means negligible, following blood normalization by means of blood withdrawal and O_2_ carrying capacity reduction via CO rebreathing in men. Although CO might increase the affinity of Hb for O_2_, it should be noted that the decrease in estimated peak arteriovenous O_2_ difference following blood normalization in men (–13.0%) coincided with the change in effective Hb (–13.0%) (Table 2), thus supporting the reduction in blood O_2_ content as the main experimental effect. While these results concur with the initial hypothesis, the striking contribution of blood variables to the sex dimorphism in VO_2p*eak*_ might not be generally expected. Certainly, blood is not the only variable along the O_2_ transport and utilization chain known to possess sex-specific characteristics (Sheel et al., 2016; Montero et al., 2018; Diaz-Canestro and Montero, 2020). In this respect, the present findings underline that not every sex difference along the O_2_ cascade, from the lungs to the mitochondria in skeletal muscle, primarily determines the gap in VO_2p*eak*_ between young women and men.

Notwithstanding the straightforward effects of blood normalization on sex differences in aerobic capacity, the impact on certain cardiovascular outcomes merits further attention. As illustrated in Figure 1, LV volumetric determinants of cardiac pumping capacity during incremental exercise did not differ between women and men after blood normalization. Yet, a trend is noted for smaller effects on LVEDV and SV at the highest exercise intensity. Indeed, Q_*peak*_ was reduced in men after blood normalization but remained increased compared with women. Similar, but of greater magnitude, effects of blood normalization on cardiac filling and pumping capacity were noticed in a previous older cohort (Diaz-Canestro et al., 2021). In this older cohort, sex differences in LVEDV, SV, and Q were mostly unaltered by the experimental matching of BV and O_2_ carrying capacity throughout the incremental exercise (Diaz-Canestro et al., 2021). Thus, additional factors besides decreased systemic blood flow must contribute to eliminating sex differences in VO_2p*eak*_ after blood normalization in both young and older individuals. In the latter, men exhibited a marked and stable decrease in cardiac afterload during exercise, as represented by lower SBP, after blood normalization (Diaz-Canestro et al., 2021). Such hemodynamic alteration might be attributed to the peripheral (compensatory) vasodilation induced by the relative state of anemic hypoxia that follows the reduction of blood O_2_ carrying capacity and arterial O_2_ content (Malo et al., 1984; Gonzalez-Alonso et al., 2001). Higher histamine production during hypoxic exercise may additionally contribute to increased vasodilation (Ely et al., 2020). In this respect, *extra* peripheral vasodilation via the infusion of vasodilators in the femoral artery is known to facilitate SV and thereby enhance cardiac pumping capacity during cycling exercise (Calbet et al., 2006; Gonzalez-Alonso et al., 2008; Bada et al., 2012). Accordingly, when the blood of older men is manipulated to have the O_2_ carrying capacity of age-matched women, the concomitant decrease in peripheral vascular tone and cardiac afterload may contribute to preserving Q throughout the incremental exercise (Diaz-Canestro et al., 2021). While seemingly paradoxical, such an O_2_-dependent peripheral vasodilation blunts O_2_ extraction in exercising limbs (Calbet et al., 2006). This could be the collateral consequence of altered blood flow distribution induced by the vasodilation of arterioles irrigating inactive muscle fibers and non-muscular tissues, which under normoxic exercise are mainly influenced by sympathetic-mediated vasoconstriction—an indispensable adjustment in order to optimize the limited capacity to deliver blood in the systemic circulation during exercise, notably with aging (Calbet et al., 2006; Lundby et al., 2008; Hearon and Dinenno, 2016). Whether the above physiological construct can be extrapolated to the present cohort including young individuals remains speculative. Indeed, the reduction in SVR during incremental exercise was attenuated in young men after blood normalization (matching with women’s levels), indicating that increased peripheral vasoconstriction plausibly induced by hypovolemia prevailed over compensatory vasodilation. Likewise, the herein substantially lesser magnitude of cardiac function preservation after blood normalization implies the influence of aging. At a young age, the intrinsically higher compliance of the heart could entail greater sensibility to changes in filling pressures, resulting in larger effects of blood withdrawal on LVEDV and thereby SV (Fujimoto et al., 2012). Additionally or alternatively, the firmly established increase in baseline vasoconstrictor signaling with aging may leave more “room” for vasodilatory responses in young individuals exposed to the combination of reduced arterial O_2_ content and blood withdrawal (Hearon and Dinenno, 2016). Further complementary research is needed to ascertain the role of peripheral mechanisms potentially modulating convective and diffusive components of O_2_ transport and therefore contributing to sex differences in cardiorespiratory fitness in young and older populations.

The final goal of any domain of physiology is to elucidate how a given function works, thus empowering to control outcomes as desired. The comprehension of the mechanisms that explain the generally lower exercise capacity of women relative to similarly trained men might open new avenues for the discovery of effective interventions, with the aim to improve performance and health outcomes in female and male populations (Lundby and Robach, 2015). In this respect, VO_2p*eak*_ is both a crucial determinant of endurance performance as well as a strong prognostic marker of cardiovascular and all-cause mortality (Kodama et al., 2009; Lundby and Robach, 2015). With respect to the major impact of VO_2p*eak*_ on exercise capacity, the question arises as to whether women and men might have similar potential to perform in endurance events if only BV and O_2_ carrying capacity were matched (Diaz-Canestro and Montero, 2020). Of note, recent studies have demonstrated higher mitochondrial content and oxidative capacity in skeletal muscle of healthy young women compared with men matched by age, VO_2p*eak*_ and running performance (Cardinale et al., 2018; Montero et al., 2018; Diaz-Canestro and Montero, 2020). Hence, women may indeed possess certain metabolic advantages at the lower end of the O_2_ transport and utilization chain (Diaz-Canestro and Montero, 2020). Notwithstanding, whether women could outperform men with a similar capacity to deliver O_2_ has been suggested in long-distance races but remains to be ascertained (Speechly et al., 1996; Montero et al., 2018; Diaz-Canestro and Montero, 2020). What can be certainly inferred thus far is that the fundamental determinants of endurance performance in men are readily amenable to modification, this being through specific training, hemodynamic or pharmacological stimuli targeting BV and O_2_ carrying capacity (Montero et al., 2016d; Montero and Lundby, 2018; Lundby and Montero, 2019). The present findings may have broader implications for health outcomes. In this regard, women generally present a ∼10% lower blood O_2_ carrying capacity than men, virtually throughout the adult lifespan (Murphy et al., 2010; Murphy, 2014). While blood Hb concentration is a strongly regulated variable mainly unresponsive to typical lifestyle interventions (Lundby and Montero, 2019), the key pathways regulating erythropoiesis are well established and thus potentially targeted by novel lifestyle and/or pharmacological strategies (Montero et al., 2016d; Montero and Lundby, 2018, 2019). Nonetheless, experimental studies have first to confirm the potential acute and long-term benefits of increasing the capacity to deliver O_2_ in women (Lundby and Montero, 2019). Ultimately, physiology, via the progressive disclosure of how the human body works, may contribute to keep questioning ingrained notions about women and men’s physical capacities.

### Limitations

Healthy individuals were selected in order to exclude the potential influence of disease-specific confounding factors. The potential influence of the menstrual phase on blood flow distribution during exercise, venous compliance, and body composition (in particular muscle mass) remains to be elucidated. In addition, potential unsought effects of –50 mmHg LBNP on central hemodynamics during acute leg cycling exercise, if any, remain to be assessed with invasive measures of ventricular filling pressures. Furthermore, the second testing (blood normalization) session was not performed in women. Consequently, the potential influence of ordering effects was not controlled. Finally, the selected experimental approach takes female BV and O_2_ carrying capacity as the “control” variables. Further studies might attempt to reassess the present findings using the inverse approach, i.e., expanding female BV and increasing O_2_ carrying capacity to match with male counterparts, if possible using a randomized double-blind cross-over design.

## Conclusion

In conclusion, blood normalization in healthy young men and women abolishes sex differences in cardiac and aerobic capacities. The fundamental role of definite blood variables such as BV and O_2_ carrying capacity in determining sex differences in cardiorespiratory fitness is herein experimentally demonstrated, in a circulatory system not altered by aging or disease.

## Data Availability Statement

The original contributions presented in the study are included in the article/Supplementary Material; further inquiries can be directed to the corresponding author/s.

## Ethics Statement

The studies involving human participants were reviewed and approved by the Conjoint Health Research Ethics Board (REB18-1654) of the University of Calgary. The patients/participants provided their written informed consent to participate in this study.

## Author Contributions

DM: conception and design of the experiments. CD-C, BP, AS, and DM: data collection, analysis, interpretation, and drafting the article or revising it critically for important intellectual content. All authors contributed to the article and approved the submitted version.

## Conflict of Interest

The authors declare that the research was conducted in the absence of any commercial or financial relationships that could be construed as a potential conflict of interest.

## Publisher’s Note

All claims expressed in this article are solely those of the authors and do not necessarily represent those of their affiliated organizations, or those of the publisher, the editors and the reviewers. Any product that may be evaluated in this article, or claim that may be made by its manufacturer, is not guaranteed or endorsed by the publisher.
