# Supplementary material for: Differences in Cardiac Output and Aerobic Capacity Between Sexes Are Explained by Blood Volume and Oxygen Carrying Capacity
Source: Front Physiol. 2022 Mar 17;13:747903. doi: 10.3389/fphys.2022.747903 (PMC8970825; doi:10.3389/fphys.2022.747903)
Supplement: Supplementary file 1 [file Data_Sheet_1.docx]

**Supplemental Material**

**Supplemental Figure 1.** Illustration of the lower body negative pressure (LBNP) chamber specifically designed for exercise testing and echocardiography.

**Supplemental Figure 1.** Illustration of the lower body negative pressure (LBNP) chamber specifically designed for exercise testing and echocardiography.

**
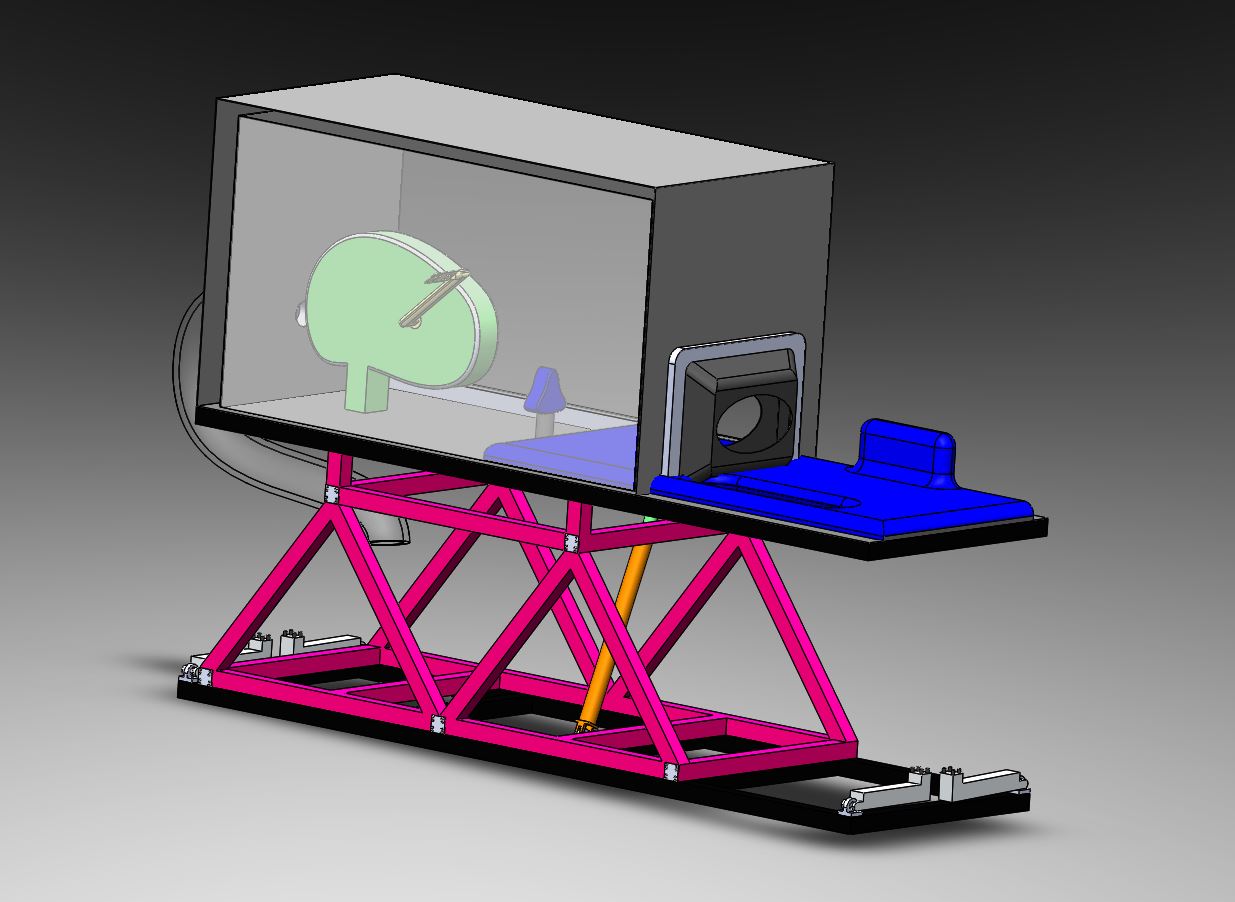
**
